# Prodigiosin: unveiling the crimson wonder – a comprehensive journey from diverse bioactivity to synthesis and yield enhancement

**DOI:** 10.3389/fmicb.2024.1412776

**Published:** 2024-06-05

**Authors:** Yonglin Lu, Derun Liu, Renhui Jiang, Ziyun Li, Xueyan Gao

**Affiliations:** ^1^Medical Science and Technology Innovation Center, Shandong First Medical University and Shandong Academy of Medical Sciences, Jinan, China; ^2^Jinan Vocational College of Nursing, Jinan, China; ^3^Department of Biostatistics, School of Public Health, Cheeloo College of Medicine, Shandong University, Jinan, China

**Keywords:** Prodigiosin, *Serratia marcescens*, bioactivity, high-level synthesis, biosynthesis

## Abstract

Prodigiosin (PG) is a red tripyrrole pigment from the prodiginine family that has attracted widespread attention due to its excellent biological activities, including anticancer, antibacterial and anti-algal activities. The synthesis and production of PG is of particular significance, as it has the potential to be utilized in a number of applications, including those pertaining to clinical drug development, food safety, and environmental management. This paper provides a systematic review of recent research on PG, covering aspects like chemical structure, bioactivity, biosynthesis, gene composition and regulation, and optimization of production conditions, with a particular focus on the biosynthesis and regulation of PG in Serratia marcescens. This provides a solid theoretical basis for the drug development and production of PG, and is expected to promote the further development of PG in medicine and other applications.

## 1 Introduction

Prodigiosin (PG), a red pigment with a core structure of tripyrrole rings, represents a secondary metabolite derived from certain sources, including *Serratia marcescens*, actinomycetes, and marine bacteria. This metabolite exhibits a wide range of biological activities, including anticancer, antimicrobial, antibiofilm, antiparasitic, and antiviral properties. In oceanography, PG has demonstrated effectiveness as an algaecide against harmful algal blooms (HABs) (Zhang et al., 2020). Moreover, in the realm of food production, PG finds utility as antioxidants, additives, colorants, and more (Arivizhivendhan et al., 2018; Sajjad et al., 2018). Significantly, extensive research has been dedicated to exploring the anticancer potential of PG and its derivatives in treating various human cancers, such as small cell lung cancer (Langer et al., 2014) and breast cancer (Wang et al., 2016). With its favorable properties, PG has garnered significant attention across diverse fields.

Within the extensive prodiginine family, PG holds the distinction of being the first discovered and most widely recognized member. Other prodiginine family members, including cycloprodigosin, undecylprodigiosin (UP), metacycloprodigiosin, and streptorubin B, exhibit distinct molecular weights or structures compared to PG but share bioactive functions suitable for medical and clinical applications (Li et al., 2022).

This review provides a comprehensive description of the properties and synthesis of PG, offering insights into future perspectives and establishing a theoretical foundation for synthetic production. The primary emphasis of this review centers on elucidating the regulatory mechanisms of the biosynthetic gene cluster and optimizing production conditions for PG in *S. marcescens*, distinguishing it from other reviews on the subject.

## 2 Structures and properties of prodigiosin

### 2.1 Structure of prodigiosin

PG is a molecule with a red linear tripyrrole structure, which concludes A, B, and C rings. The three rings are pyrrole, 3-methoxypyrrole, and 2-methyl-3-pentylpyrrole, respectively. Two of the rings, A and B, are linked together to form a bipyrrole structure, and the C ring is connected to the B ring via a methylene group. The structures of some members of prodiginine family are shown in Table 1.

**Table 1:** Structures, sources, and bioactivities of some major PGs.

Name	Structure	Bacterial species	Bioactivity	Reference
Prodigiosin	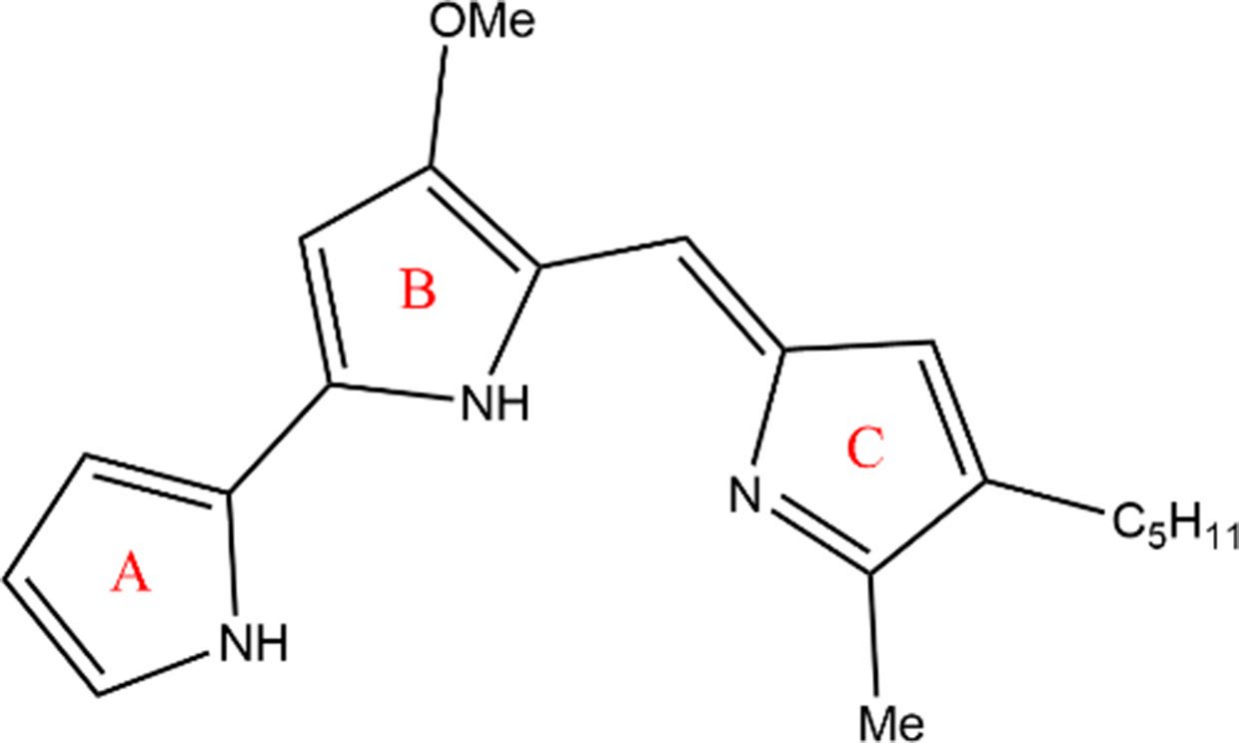	*Serratia marcescens* *Serratia rubidaea* *Serratia* 39,006 *Streptomyces coelicolor* *Streptomyces griseoviridis* *Vibrio spartinae* *Janthinobacterium lividum* *Hahella chejuensis* *Pseudoalteromonas rubra* *Zooshikella rubidus* *Massilia frigida*	Anticancer Antibacterial Antifungal Antialgal Antimalarial Antibiofilm Antioxidant Antiaging Immunosuppressive	Williamson et al. (2006), Kim et al. (2007), Kawasaki et al. (2009), Schloss et al. (2010), Setiyono et al. (2020), Vitale et al. (2020), Sun et al. (2021), Shaffer et al. (2023), Ma et al. (2024)
Undecylprodigiosin	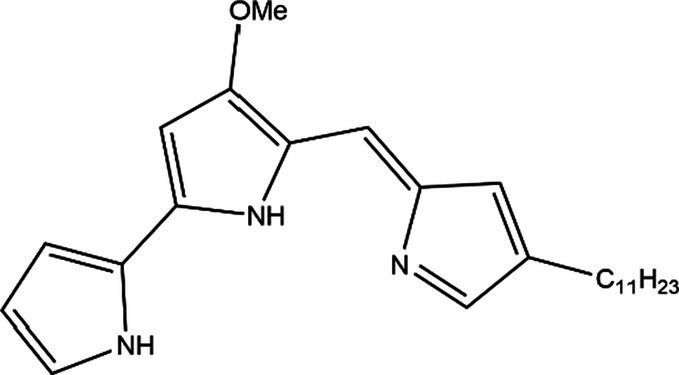	*Serratia marcescens* *Streptomyces coelicolor* *Streptomyces longispororuber* *Hahella*	Anticancer Antibacterial Antifungal Antimalarial Antialgal Immunosuppressive	Williamson et al. (2005), Kim et al. (2007), Stankovic et al. (2014)
Decylprodigiosin	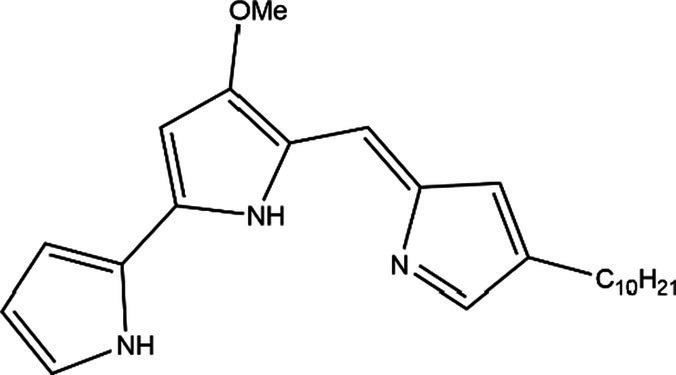	*Streptomyces violaceoruber*	Anticancer Antimicrobial	Girão et al. (2024)
Cycloprodigiosin	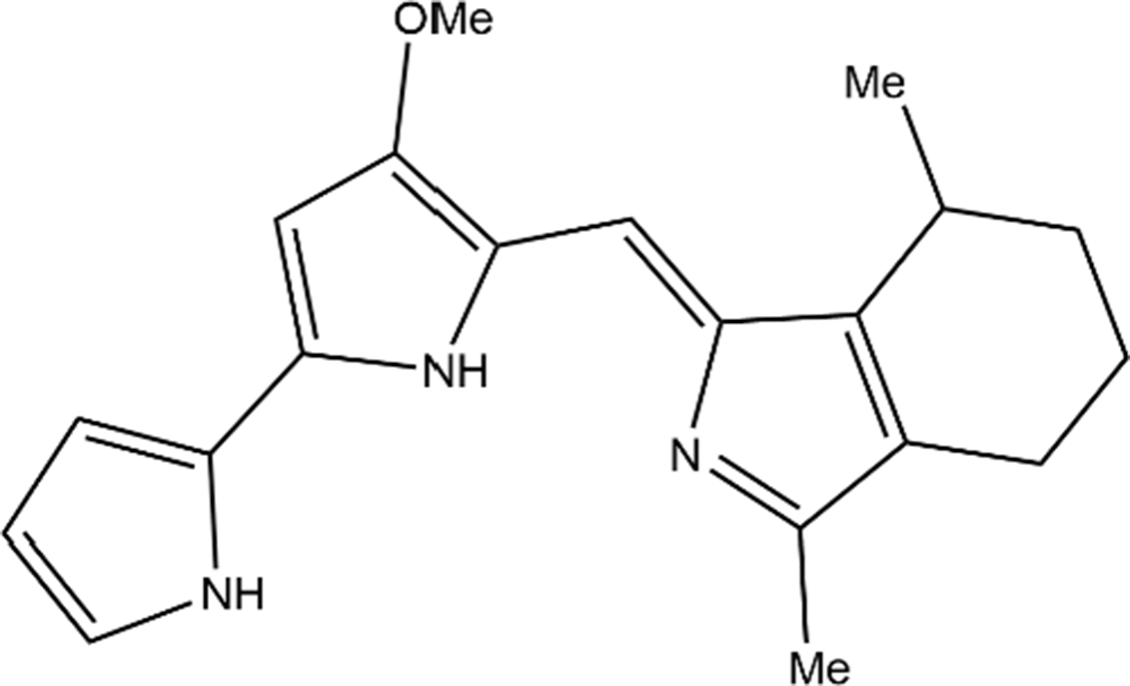	*Pseudoalteromonas rubra* *Zooshikella rubidus* *Massilia frigida* *Vibrio spartinae* *Vibrio gazogenes*	Anticancer Antibacterial Antifungal	Setiyono et al. (2020), Shaffer et al. (2023)
Metacycloprodigiosin	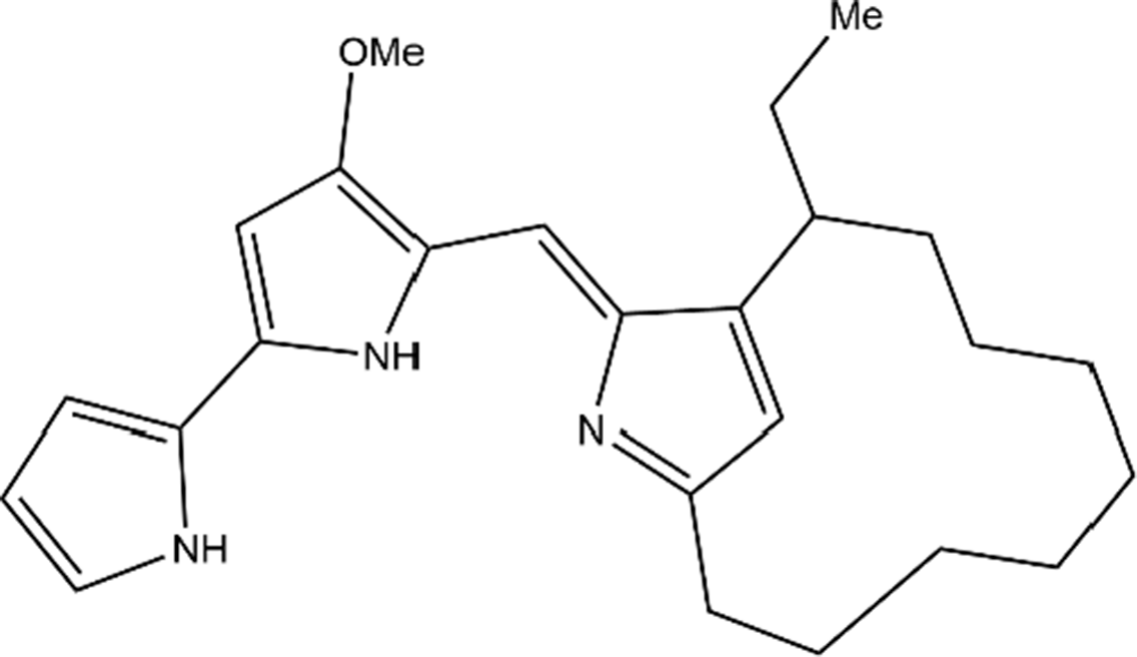	*Streptomyces longispororuber*	Anticancer Antimalarial Immunosuppressive	Wasserman et al. (1969), Ikeda et al. (2016)
Streptorubin B	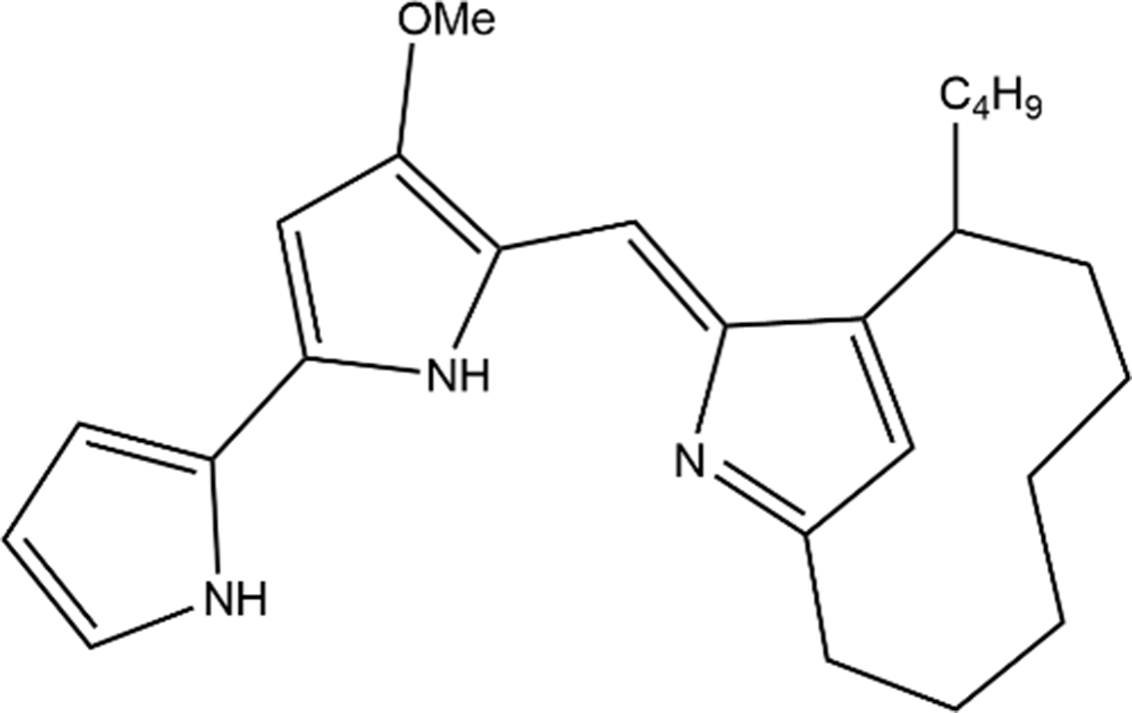	*Streptomyces coelicolor*	Antibacterial Antimalarial	Yi et al. (2022)
Prodigiosin R1	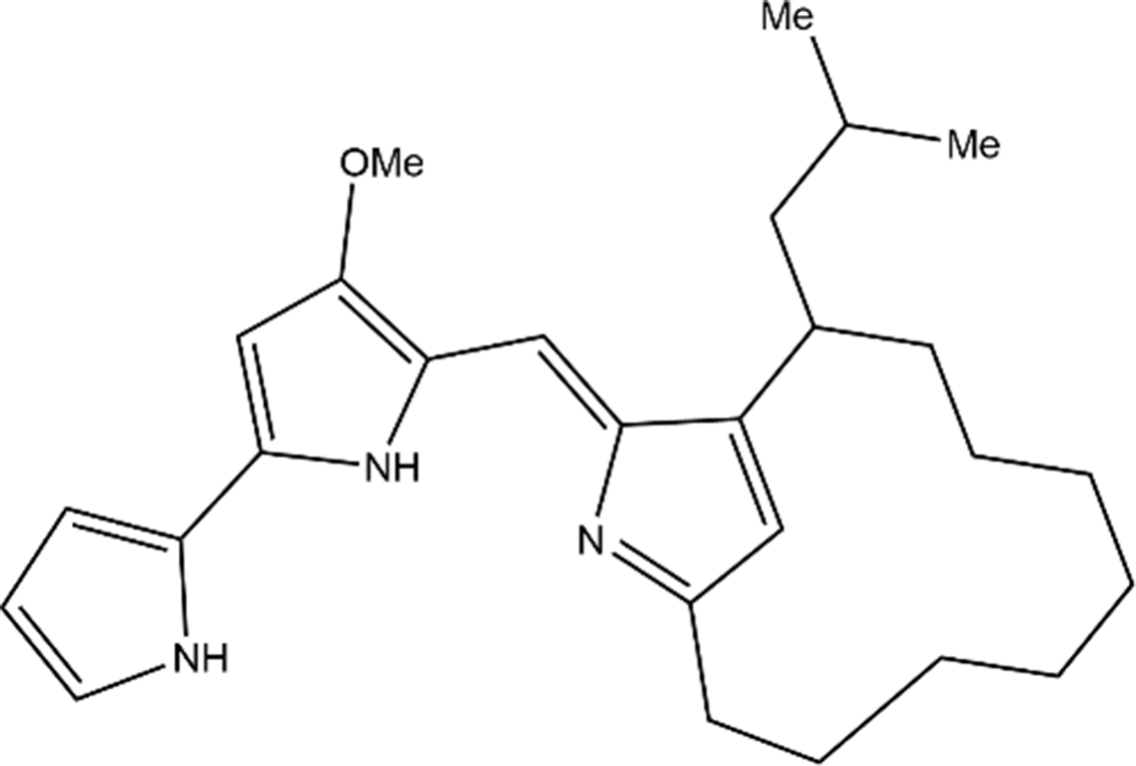	*Streptomyces griseoviridis*	Pending Research	Kawasaki et al. (2008)
Prodigiosin R2	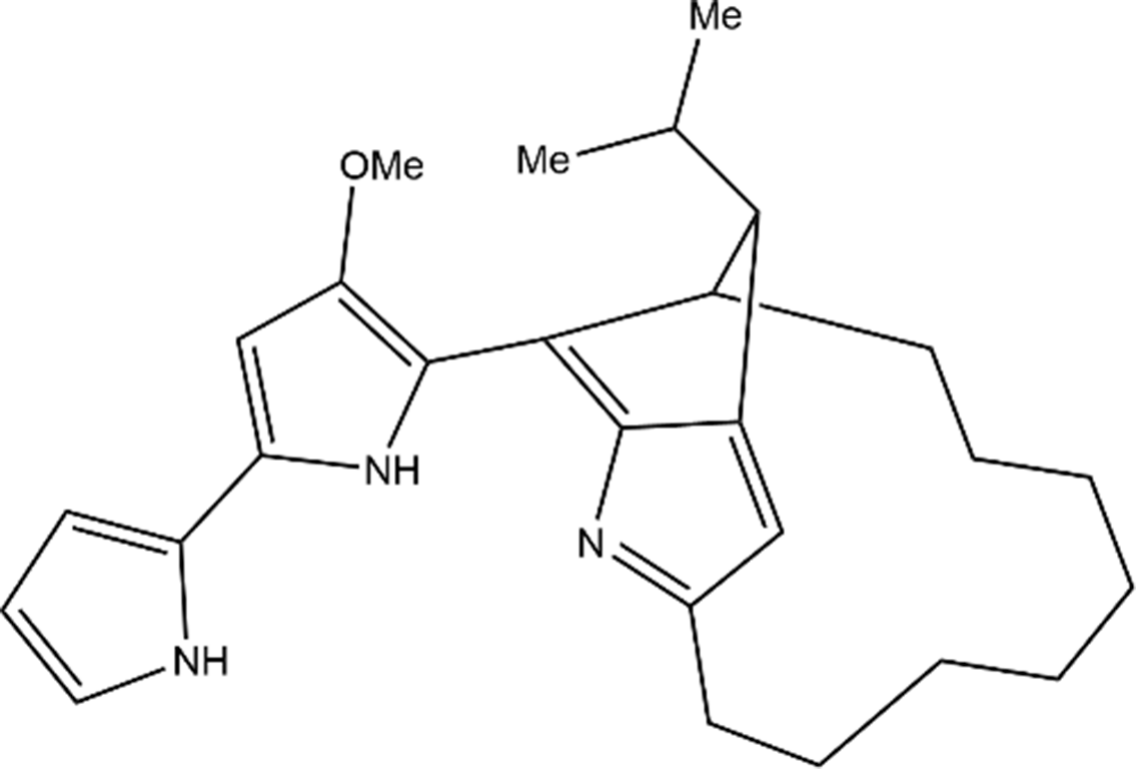	*Streptomyces griseoviridis*	Anticancer	Kimata et al. (2018)
Prodigiosin R3	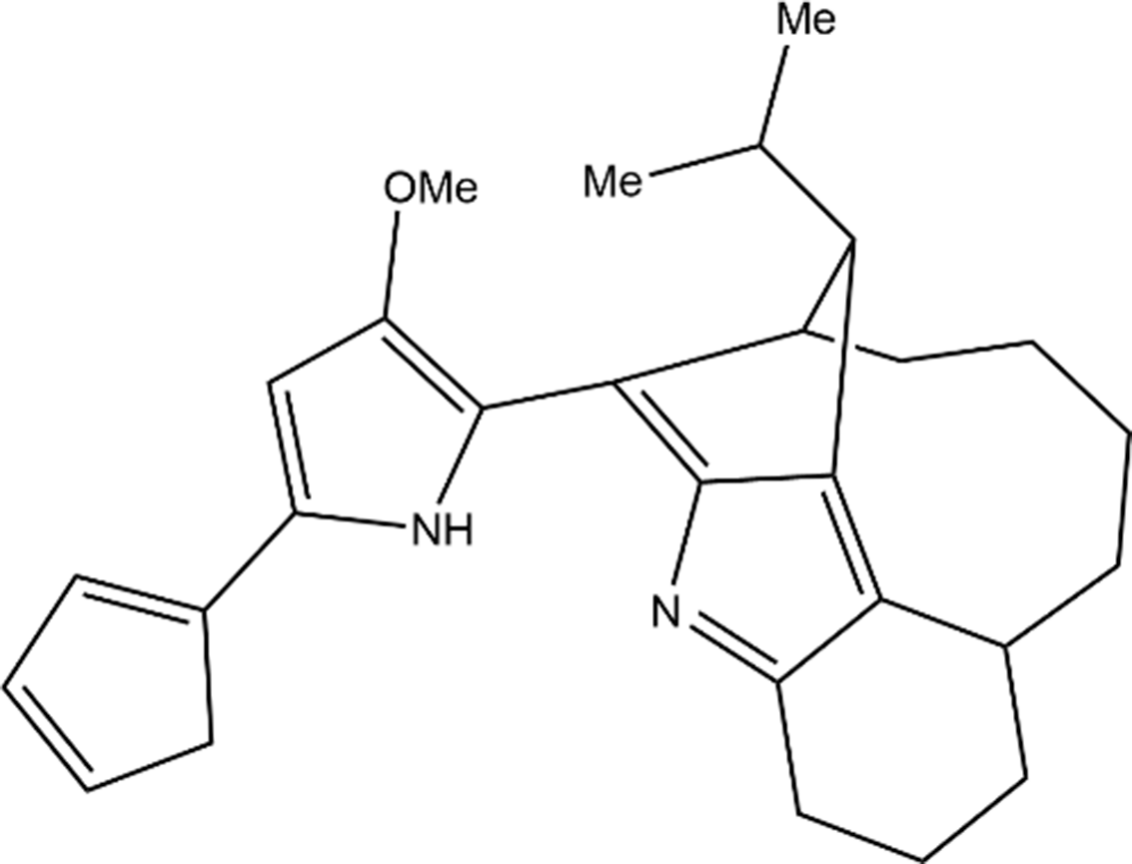	*Streptomyces coelicolor*	Anticancer	Kimata et al. (2022)

In general, prodiginine can be divided into two main parts: linear and cyclic prodiginine. The main difference in linear prodiginine is the length of the carbon chain of the substituent group in the C-ring. The cyclic prodiginine can be divided into two subgroups, one of which forms a ring on the C-ring, such as cycloprodigiosin and Streptorubin B; Another subgroup is connecting the A ring with the C ring to form a ring, such as cyclononylprodigiosin and methylcyclooctylprodigiosin (Hu et al., 2016).

### 2.2 Physical properties of prodigiosin

The physical properties of PG play a crucial role in its application and identification during production. Despite being insoluble in water, PG exhibits moderate solubility in ether and alcohol, and can be dissolved in chloroform, methanol, acetonitrile, and dimethyl sulfoxide. The sensitivity of PG to light poses challenges in commercial dye applications (Someya et al., 2004). Spectrophotometric analysis under different pH conditions reveals that PG can manifest as either red (with a sharp peak at 535 nm) in acidic media or orange-yellow (with a broad curve centered on 470 nm) in alkaline environments (Jehlička et al., 2021). However, since PG analogs also have similar absorbance, this information is not sufficient for PG identification, so more sophisticated assays are required, such as the GC–MS, LC–MS or characteristic bands using RAMAN spectroscopy. Mass spectrometry analysis revealed that the mass-to-charge ratio(m/z) of PG was 324.2([M + H^+^]), and the molecular weight of PG is 324 Da. Rf values ranging from 0.9 to 0.95 are a distinctive feature of PG (Song et al., 2006; Bhagwat and Padalia, 2020).

### 2.3 Bioactivity of prodigiosin

#### 2.3.1 Anticancer activity

Cancer, a non-communicable disease, is a major global cause of death, claiming nearly 10 million lives in 2020, making up about one-sixth of all deaths. Predominant cancers include breast, lung, colon, rectum, and prostate, with lung, colon, rectal, liver, stomach, and breast cancers showing the highest mortality rates (Sung et al., 2021). Effective cancer therapies are crucial for human health, with natural compounds being closely studied for medicinal potential.

PG has shown strong anti-cancer effects, with proven efficacy in treating melanoma, breast cancer, lung cancer, lymphocytic leukemia, and glioblastoma (Pan et al., 2012; Brown et al., 2015; Wang et al., 2016; Cheng et al., 2018; Chiu et al., 2018; Espona-Fiedler et al., 2022). It triggers apoptosis in cancer cells, including multidrug-resistant ones, with minimal toxicity to normal cells (Elahian et al., 2013). Obatoclax, a prodigiosin derivative, displays potent anti-cancer and pro-apoptotic properties. Clinical trials have demonstrated its effectiveness in treating relapsed chronic lymphocytic leukemia and extensive-stage small cell lung cancer, either alone or in combination with other drugs, resulting in positive therapeutic outcomes (Langer et al., 2014; Brown et al., 2015).

PG’s anti-cancer effects involve diverse mechanisms, with a key role played by promoting apoptosis in tumor cells. Apoptosis can be initiated through three pathways: the extrinsic (death receptor) pathway, the intrinsic (mitochondrial) pathway, and the endoplasmic reticulum (ER) pathway. PG primarily influences cellular apoptosis via the intrinsic (mitochondrial) pathway, which is regulated by the Bcl-2 protein family comprising both pro-apoptotic (Bax, Bak, Bid) and anti-apoptotic (Bcl-2, Bcl-W, Mcl-1) proteins. The balance between these proteins determines the initiation of cellular apoptosis (Wong, 2011). PG and Obatoclax bind to the BH3 domain of anti-apoptotic proteins, shifting the balance toward cell death by affecting the pro- and anti-apoptotic proteins. Experimental evidence in melanoma cells shows that PG disrupts the Mcl-1/Bak complex, leading to Bak release and activating the intrinsic apoptotic pathway (Hosseini et al., 2013). PG also induces apoptosis by altering pH levels, DNA cleavage, cell cycle arrest, and other mechanisms (Darshan and Manonmani, 2015).

**Figure 1:**
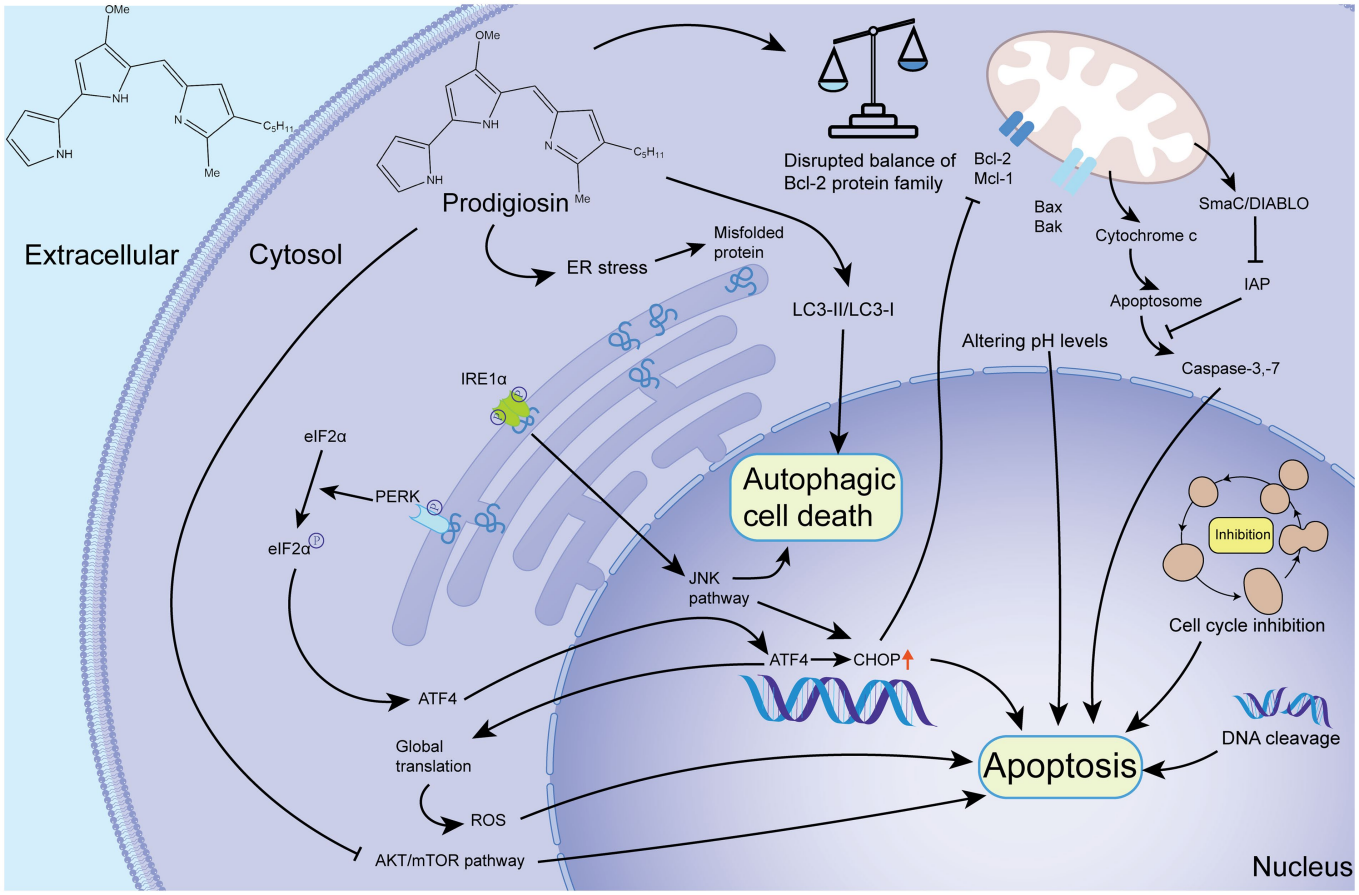
Schematic diagram of the mechanism of PG-mediated apoptosis and autophagic cell death. Upon entering the cell, PG can induce apoptosis by disrupting the balance of Bcl-2 family proteins and initiating the mitochondrial pathway. It can also induce apoptosis through the alteration of pH, cell cycle inhibition, and DNA cleavage. Additionally, PG can cause ER stress and autophagic cell death and apoptosis through the IRE1α-JNK and PERK-eIF2α-ATF4-CHOP pathway. The ATF4-CHOP pathway is responsible for autophagic cell death and apoptosis. In addition to the aforementioned pathways, PG can inhibit the AKT/mTOR pathway to cause apoptosis. Furthermore, PG can affect LC3 protein, which indicates that PG can cause autophagic cell death. IAP, inhibitor of apoptosis protein; ROS, reactive oxygen species; Smac, second mitochondrial-derived activator of caspases; DIABLO, director inhibitor of apoptosis-binding protein with Low pI (O’Brien and Kirby, 2008; Wong, 2011).

Research on the ER pathway of PG has revealed its role in inducing ER stress, which can trigger cell apoptosis through the IRE1α-JNK and PERK-eIF2α-ATF4-CHOP axes (Wang and Kaufman, 2016). PG induces ER stress by downregulating Bcl-2 and activating the PERK-eIF2α-ATF4-CHOP pathway or the IRE1α-JNK pathway in the ER (Pan et al., 2012). Furthermore, the ER pathway also contributes to autophagic cell death, as demonstrated in a study on human glioblastoma cells where PG activated the JNK pathway and inhibited the AKT/mTOR pathway, leading to autophagic cell death through ER stress (Cheng et al., 2018). A schematic of the mechanisms involved in apoptosis and autophagic cell death is shown in Figure 1.

Inhibiting tumor cell migration and motility is essential for achieving anti-cancer effects. Research by Margarita Espona-Fiedler and her team found that PGs effectively reduce the adhesive capability of melanoma cells, impeding their ability to colonize distant organs and preventing metastasis (Espona-Fiedler et al., 2022). PG treatment also decreases the adhesive capacity of metastatic lung cancer cells and reduces filopodia formation in melanoma cells, which are crucial for cancer cell migration and proliferation. Furthermore, PG treatment downregulates genes associated with migration and invasion, such as Twist-1, MMP-1, MMP-2, and RhoA (Zhang et al., 2005).

#### 2.3.2 Antimicrobial activity

Bacterial hazards have far-reaching impacts on human health, agriculture, the environment, and food safety, leading to infectious diseases, agricultural losses, environmental pollution, and antibiotic resistance. For instance, *Staphylococcus aureus* can result in food poisoning; *Candida albicans* can cause oral mucosal disease. It is essential to control the spread of antibiotic resistance to protect health, the environment, and food safety. Misuse of antibiotics has made treating infectious diseases difficult, emphasizing the need for new antibiotic discovery and development.

**Figure 2:**
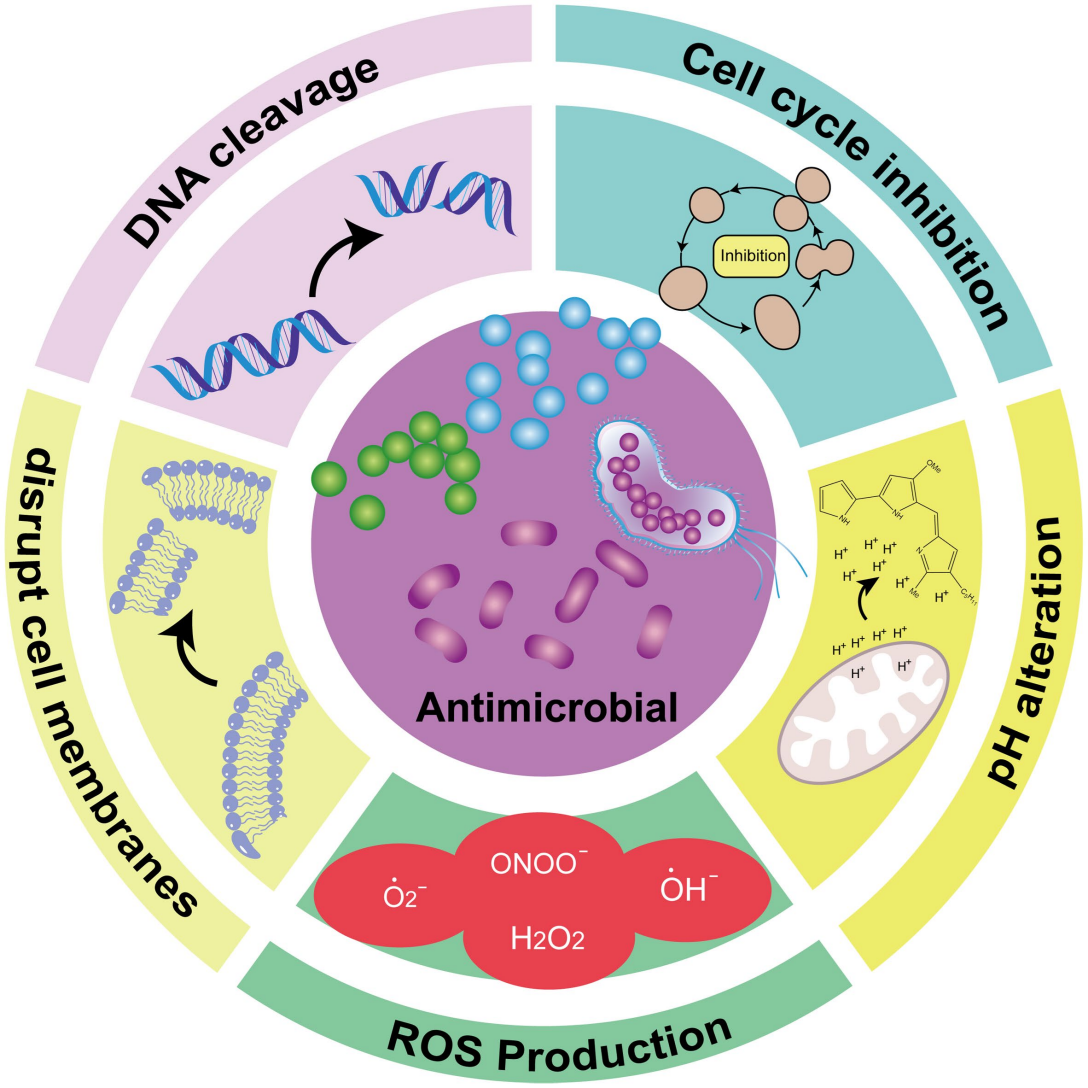
Schematic diagram of the PG antimicrobial mechanism (Francisco et al., 2007).

PGs exhibit broad-spectrum antimicrobial activity against a wide range of bacteria and fungi, including *Enterococcus faecalis*, *Staphylococcus aureus*, *Streptococcus pyogenes*, *Salmonella typhimurium*, *Escherichia coli*, *Bacillus subtilis*, and *Candida albicans* (Lapenda et al., 2015). However, compared to Gram-positive bacteria, PG’s inhibitory effects on Gram-negative bacteria are less pronounced. Research shows that cyclic PGs offer superior antibacterial effects compared to linear PGs (Lee et al., 2011). The lipophilic nature of PGs allows them to disrupt cell membranes through various mechanisms, leading to bacterial death (Ravindran et al., 2020). Additional antimicrobial properties of PGs include pH alteration, DNA cleavage, cell cycle inhibition, and induction of reactive oxygen species (ROS) production (Yip et al., 2019; Choi et al., 2021) (Figure 2). Due to this antimicrobial property, PG has been designed for use in food packaging. Amorim et al. added PG to the outer and inner layers of food packaging materials with good results, although it should be noted that PG is sensitive to light, and the stability of the outer layer under light conditions deserves to be studied (Amorim et al., 2022).

#### 2.3.3 Anti algae bloom

HABs pose a threat to aquatic ecosystems, marine life, and human health due to the toxins they produce. These blooms, fueled by nutrient overload, can result in oxygen depletion, fish kills, and disruptions in marine food chains. The economic impact includes losses in fisheries and tourism from beach closures and water quality problems. Effective monitoring and management strategies are crucial to mitigate these adverse effects. PG demonstrates effectiveness in controlling various algae species, including *Prorocentrum donghaiense* (Chen et al., 2021), red tide dinoflagellates (Jeong et al., 2005), *Heterosigma akashiwo* (Zhang et al., 2020), *Phaeocystis globosa* (Zhang et al., 2016), and *Microcystis aeruginosa* (Zhang et al., 2022), without harming non-target organisms such as fish. Its mechanism of action involves inducing ROS production and lipid peroxidation while inhibiting the cell cycle of harmful algae. This targeted approach makes PG a practical and effective solution for HAB mitigation (Zhang et al., 2016; Chen et al., 2021).

## 3 Synthesis of prodigiosin

PG can be produced through biosynthesis or chemical synthesis. Biosynthesis occurs inside specific bacteria like *Serratia* and *Streptomyces*, utilizing enzymes and genes for a natural and sustainable process suitable for large-scale production. In contrast, chemical synthesis involves a more complex multi-step organic process, offering precise control over the PG structure but potentially higher costs and operational complexity. When choosing a synthesis method, factors to consider include production scale, cost-effectiveness, and efficiency. This article provides an in-depth examination of PG biosynthesis for practical applications (Hu et al., 2016).

### 3.1 Biosynthesis of prodigiosin

The biosynthesis of PG was first articulated in 1966 and PG was synthesized by enzymatic condensation reaction of 2-methyl-3-pentylpyrrole (MAP) and 4-methoxy-2,2′-bipyrrole-5-carbaldehyde (MBC) (Morrison, 1966). Williams also revealed that PG’s precursors are derived from a number of amino acids and acetates. Among them, Wasserman’s study confirmed that MAP is derived from 2-octenal and pyruvate while MBC biosynthesis starts from L-proline and malonyl coenzyme A (Wasserman et al., 1973; Williams, 1973).

In the biosynthesis of PG, certain peptide products are produced via nonribosomal peptide synthesis (NRPSs) rather than ribosomes. NRPSs function by sequentially adding substrates to the peptide chain through a multienzyme complex utilizing the thiotemplate mechanism. These NRPSs comprise three key structural domains: adenylation domains for substrate selection and activation to amide-AMP, peptidyl carrier protein (PCP) domains for immobilizing activated substrates as thioesters to 4′-phosphopantetheine (Ppant), and condensation domains catalyzing the formation of peptide bonds between donor polypeptides and acceptor amino acids (Süssmuth and Mainz, 2017).

#### 3.1.1 Genes responsible for PG synthesis

**Figure 3:**
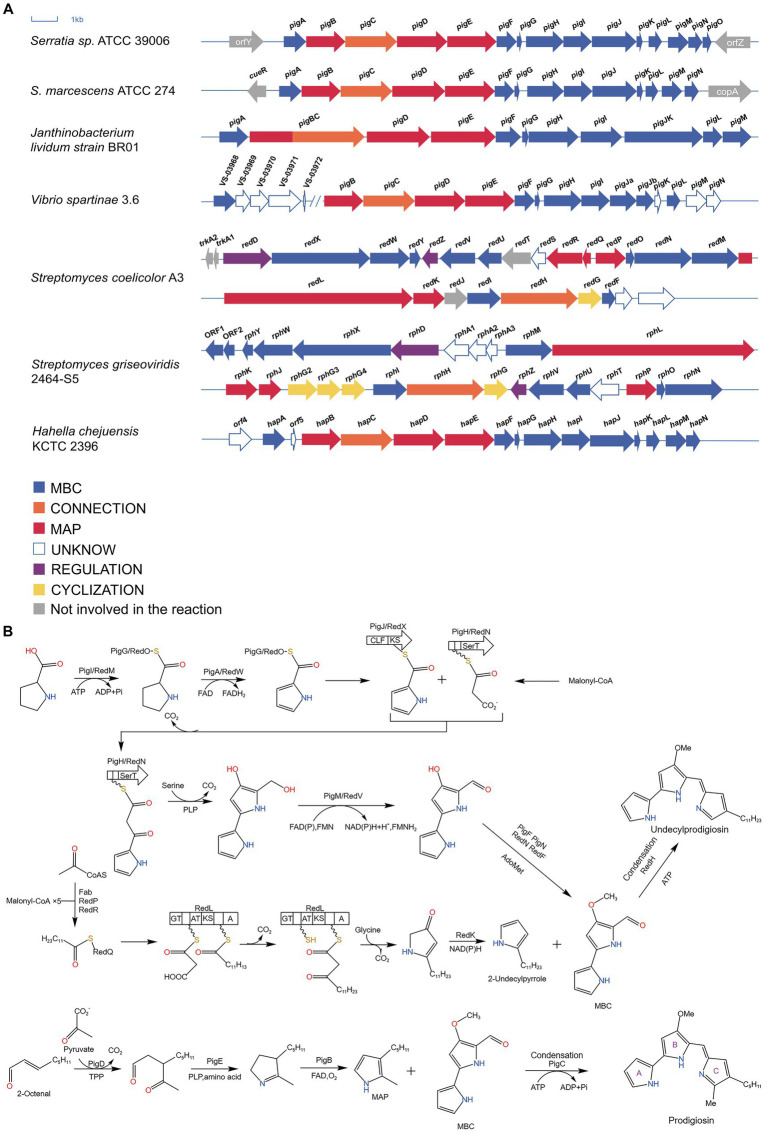
Biosynthesis of prodigiosin. **(A)** Clusters of genes related to the synthesis of PG in several microorganisms. Blue and red arrows indicate genes that synthesize MBC and MAP, respectively; orange arrows indicate genes that link MBC to MAP; yellow arrows indicate genes involved in ring formation; purple arrows indicate genes involved in regulation; and white and gray arrows indicate genes not involved in the synthesis reaction and unknown genes, respectively (Cerdeno et al., 2001; Harris et al., 2004; Kim et al., 2006; Kawasaki et al., 2009; Schloss et al., 2010; Vitale et al., 2020). **(B)** The biosynthetic pathway of PG (Williamson et al., 2006; Kim et al., 2007).

In 2004, Harris and his team cloned, sequenced, and expressed microorganisms that produce PG in heterologous hosts, including *S. marcescens* ATCC 274 (Sma274) and *Serratia* sp. ATCC 39006 (S39006). (Harris et al., 2004). Subsequently, *Hahella chejuensis* KCTC 2396 and *Janthinobacterium lividum* strain BR01 (JliBR01) were sequenced in 2006 and 2010, respectively, (Kim et al., 2006; Schloss et al., 2010). In 2020, through genomics and metabolomics, a new branch of PG production was discovered in marine *Vibrio spartinae* 3.6 (Vitale et al., 2020). The various microorganisms described above all contain 13–15 genes related to PG, while two *Streptomyces* species, *Streptomyces coelicolor* A3 and *Streptomyces griseoviridis* 2464-S5, have more complicated homologous gene clusters (Cerdeno et al., 2001; Kawasaki et al., 2009). The relevant gene clusters are shown in Figure 3.

#### 3.1.2 Biosynthesis of MAP

2-octenal, derived from the production of fatty acid biosynthesis enzymes or the autoxidation of unsaturated fatty acids, is a precursor for MAP synthesis. It reacts with pyruvate to form 3-acetyloctal under the action of *pigD* and thiamine pyrophosphate (TPP). *pigD* is homologous to acetolactate synthase and can catalyze the decarboxylation of pyruvate, binding the decarboxylated two-carbon fragment to 2-Octenal; then, *pigE*, which has transamination effect, acts on 3-acetyloctal to produce aminoketones, which self-cyclize to form H_2_MAP. Finally, H_2_MAP undergoes oxidative dehydrogenation to generate MAP (Williamson et al., 2005).

#### 3.1.3 Biosynthesis of 2-undecylpyrrole

RedP condenses acetyl coenzyme A and malonyl *redQ* (ACP) to produce an acetoacetyl thioester with *redQ* attached; the carbonyl group is then reduced and dehydrated by the ketoreductase, dehydratase, and enoyl reductase of *S. coelicolor* fatty acid synthase (FAS) to produce butyryl ACP; the remaining four malonyl-*redQ* are catalyzed the subsequent extension step by *redR*, which transfers the resulting dodecyl group to the ACP structural domain of *redL*, accompanied by reduction and dehydration by FAS reductase; the other ACP attached to *redL* is then decarboxylated in the presence of ketosynthase, generating a β-ketomyristoyl thioester. The decarboxylation of β-ketomyristoyl thioester via pyridoxal phosphate (PLP) mediated condensation of glycine with an ACP-bound thioester releases CO_2_, and this intermediate is cyclized to produce 4-keto-2-undecylpyrroline. The final step is the dehydrogenation and reduction of 4-keto-2-undecylpyrroline by *redK* to yield 2-undecylpyrrole.

#### 3.1.4 Biosynthesis of MBC

*pigI/redM* activates L-proline with ATP, and then transfers it to the adjacent PCP (*pigG*/*redO*) domain to form L-prolinyl-S-PCP; L-proline-S-PCP was then oxidized and dehydrogenated to obtain pyrrole-2-carboxy-S-PCP under the action of *pigA*/*redW*. Pyrrole-2-carboxy-S-PCP was transferred from *pigG*/*redO* to cysteine with *pigJ*/*redX* active centers to obtain pyrrole-2-carboxy-S-*pigJ*/*redX*; at the same time, the malonyl group is transferred from malonyl CoA to the 4′-phosphophenylene of an ACP in *pigH*/*redN*, and then the complex decarboxylates and attacks the thioester bond of pyrrole-2-carboxy-S-*pigJ*/*redX*, generating a pyrrole-β-ketothioester attached to *pigH*/*redN*. In the presence of *pigH*/*redN* pyrrole-β-ketothioester reacts with serine to form 4-hydroxy-2,2′-bipyrrole-5-methanol (HBM). The hydroxyl group of HBM was oxidized to form an aldehyde group in the presence of *pigM*/*redV* to give 4-hydroxy-2,2′-bipyrrole-5-carbaldehyde (HBC). Finally, HBC is methylated in the presence of *pigF* and *pigN* or *redN* and *redF* to generate MBC.

#### 3.1.5 Condensation

*pigC* and can catalyze the condensation of MAP and MBC, and *redH*, a homolog of *pigC*, catalyzes the condensation of 2-undecylpyrrole and MBC to produce PG and UP, respectively. The understanding of the process of PG biosynthesis as well as the genes facilitates our use of microorganisms to synthesize PG in large quantities and to conduct research on PG.

### 3.2 Regulation of synthetic gene cluster

The transcription process of synthesizing PG is jointly regulated by multiple factors and has a relatively.

complex regulatory mechanism, including quorum sensing (QS) system, two component regulatory systems (TCSs) and various regulatory factors. The bacterial regulatory system QS, which responds to changes in bacterial density, is gaining attention. It is a communication process between bacterial cells involving the production, release, accumulation, and response to autoinducers (AI), extracellular signaling molecules (Mukherjee and Bassler, 2019). Gram-negative bacteria, like *Serratia*, utilize QS to regulate gene expression using N-acyl homoserine lactones (acyl-HSLs) as AI (AI-1) (Wei and Lai, 2006). In *Serratia* sp. ATCC 39006, PG production is regulated by QS through the LuxIR homologs SmaI/SmaR and SpnI/SpnR. SmaR inhibits the synthesis of PG by inhibiting the transcription of PG gene clusters, while SmaI can synthesize C4-HSL and bind to the transcription inhibitor SmaR. However, at low cell density, the synthesis of C4-HSL is insufficient to relieve the inhibitory effect of SmaR on transcription, and PG production is inhibited; When the cell density is high, the concentration of C4-HSL reaches the threshold and binds to SmaR, reducing the inhibitory effect and increasing PG production. A similar system is observed with SpnI/SpnR. Besides AI-1’s role in regulating PG synthesis, LuxS/AI-2 can also regulate PG synthesis. The LuxS enzyme catalyzes bacterial methyl metabolism, producing AI-2 as a metabolic byproduct. In *S. marcescens* 274, researchers have demonstrated that the PG yield significantly decreases in *luxS* mutants compared to the wild-type *S. marcescens* 274. Subsequently, through transversely expressing LuxS, it was proven that the direct cause of reduced PG production is the absence of LuxS (Coulthurst et al., 2004). Hence, AI-1 and AI-2 can be ingeniously employed in the process of PG synthesis to enhance PG yield. TCSs are composed of two proteins, namely transmembrane sensor histidine kinases (HK) and response regulatory (RR) factors, which are widely distributed in the bacterial community and have important roles in regulating intercellular communication and secondary metabolism. The basic mechanism of this system is that when HK recognizes a specific signal, its conserved histidine residue undergoes self-phosphorylation, and then the phosphate group is transferred to the conserved aspartic acid residue of RR. RR phosphorylation can promote specific gene transcription (Zschiedrich et al., 2016). The sensing kinase GacS and response regulatory factor GacA are typical TCS that are widely present in Gram negative bacteria. They regulate bacterial QS, secondary metabolism, biofilm formation, and movement through Gac/Rsm signaling pathways, including PG synthesis (Song et al., 2023). The PigQ/PigW mentioned earlier is a homolog of GacA/GacS and participates in PG synthesis. In addition, there are also EepR/EepS (Stella et al., 2015), PhoB/PhoR (Gristwood et al., 2009), RssB/RssA (Horng et al., 2010), RcsB/RcsC (Pan et al., 2021), BarA/UvrY (Liu et al., 2023), EnvZ/OmpR (Jia et al., 2022), and CpxA/CpxR (Sun et al., 2020) involved in the synthesis of PG. Among them, RssB/RssA, RcsB/RcsC and BarA/UvrY are negatively regulated and can inhibit PG synthesis; the rest are positively regulated.

In addition to these two systems, various regulatory factors can control the transcription of gene clusters. The σ factor is an important subunit in the RNA polymerase holoenzyme, and the core enzyme lacking the σ factor has almost no RNA synthesis activity, as the role of this factor is to recognize the transcription start point. RpoS is a typical σ factor in Gram-negative bacteria, and its influence on PG synthesis varies across different bacterial model experiments. In the study of *Serratia* sp. ATCC 39006, it was found that RpoS can negatively regulate the synthesis of PG; while in the study of *S. marcescens* 1912768R, RpoS can positively regulate the synthesis of PG. The specific mechanisms for these two cases are still unclear, but it can be demonstrated that the regulation of PG by RpoS is diverse. The reasons for this diversity may be attributed to differences in gene clusters or their association with QS (Wilf and Salmond, 2012; Qin et al., 2020). In addition to the σ factor, researchers have also investigated the ω factor, which also belongs to RNA polymerase. The *rpoZ* gene encodes the synthesis of the ω factor, playing a crucial role in UP synthesis and in the absence of the *rpoZ* gene, the production of UP decreases considerably, which can be partially restored by the supplementation of the *rpoZ* gene (Santos-Beneit et al., 2011).

Fumarate and nitrate reduction regulatory protein (Fnr) is a global regulatory factor of the Crp/Fnr family that controls gene transcription. At the transcriptional level, Fnr has a negative impact on the production of PG in *Serratia* sp. ATCC 39006 under aerobic conditions. There is a Fnr binding site between the −10 and − 35 regions of *pigA*, which inhibits the synthesis of PG by disrupting the binding of the promoter and RNA polymerase (Sun et al., 2021).

Stringent starvation protein A (SspA) is an RNA polymerase-associated regulatory protein. While studying Pseudoalteromonas sp. strain R3, the researchers found that strains lacking SspA are unable to produce prodiginine. The reason for this is related to the iron carrier-dependent iron uptake pathway: SspA positively regulates the expression of the siderophore biosynthetic gene (*pvd*) cluster. Its absence hinders the uptake of iron through the iron carrier-dependent pathway, leading to iron deficiency and subsequently affecting prodiginine synthesis (Yin et al., 2021).

The PtrA protein is transcribed from the *ptrA* gene, which promotes transcription of the pig gene in *S. marcescens* FZSF02. PsrA is a transcriptional regulator belonging to the LysR family. It binds directly to the promoter region of the pig operon at a regulatory binding site (RBS) and an activating binding site (ABS) in the promoter region of the pig manipulator. This positive regulation leads to an increase in PG transcription in *S. marcescens*. Both were detected by real-time quantitative PCR (RT-qPCR) and there was an increase in gene expression in *pigA*-*pigN* (Pan et al., 2022; Lin et al., 2023).

### 3.3 Factors affecting synthesis

PG is a secondary metabolite that emerges in the advanced growth stages, with its production susceptibility to an array of factors such as light, temperature, pH level, microbial characteristics, and culture medium composition. Efforts are continuously focused on investigating the most favorable production conditions and refining production techniques to enhance PG yields.

It is well known that PG is light-sensitive and demonstrates instability in illuminated environments (Someya et al., 2004). Consequently, the yield fluctuates with light intensity, resulting in a lower yield under light conditions compared to dark conditions (Ryazantseva et al., 2012). Wang et al. elucidated that PG absorbs light and causes phototoxicity on bacterial cell membranes, leading to PG leakage (Wang et al., 2013).

Temperature affects PG synthesis in two main ways. Firstly, the activity of the condensing enzyme produced by *pigC*. Lower temperatures below 22°C reduce enzyme activity, leading to decreased PG production, while higher temperatures above 30°C render the enzyme inactive, halting PG synthesis. Secondly, the *cpx* (TCS) in Gram-negative bacteria is vital in temperature-dependent PG regulation. At elevated temperatures, the sensor HK protein CpxA is activated, triggering a phosphorylation cascade that ultimately inhibits transcription of pig gene clusters and reduces PG production (Sun et al., 2020). Additionally, pH levels also impact pigment deposition, with pH 7–9 being optimal, while extremes of below 3.0 or above 10.0 are unfavorable (Sumathi et al., 2014).

The level of dissolved oxygen in the culture medium has a profound effect on PG production and agitation and aeration are important for dissolved oxygen levels (Heinemann, 1970). Research by C. Sumathi et al. demonstrated that the most optimal yield of PG was achieved at a stirring rate of 200 r.p.m. and aeration of 3 vvm. Both too high and too low rates of stirring and aeration led to decreased PG production (Sumathi et al., 2014). While previous studies suggested a high oxygen transfer rate (OTR) was beneficial for PG production, recent findings during UP synthesis indicated that lower OTRs actually increased yield. This is attributed to oxygen limitation prompting a shift from primary to secondary metabolism, ultimately enhancing production (Gamboa-Suasnavart et al., 2018). Therefore, identifying the correct stirring and aeration rates to achieve an optimal OTR is essential for maximizing PG yield. Negative feedback regulation impacts PG production in batch cultures, prompting the development of various bioreactors to improve yields. Extraction columns using HP-20 adsorbent resin demonstrated a 31% increase in pigment production compared to traditional batch fermentation methods (Kim et al., 2000; Song et al., 2006). The aforementioned optimizations for production conditions are based on *Serraria* and may not be applicable to the remaining strains that produce PG.

Researchers have harnessed genetic engineering techniques or optimized culture conditions to maximize PG production. In the realm of genetic engineering, researchers possess the ability to selectively target and modify the genes of the host cell to obtain the desired genotype.

Firstly, we can knock out or modify genes that are unfavorable for PG production or enhance the expression of genes favorable for PG production to achieve higher yields. *Serratia* sp. ATCC 39006 has a gene called *ser39006_013370* can encode a regulatory protein Fnr. Di Sun et al. inserted mini-*Tn5* transposon into *ser39006_ In 013370*, the production of PG per cell unit was higher than that of the wild-type. It is worth noting whether changing the gene structure will have an impact on the normal life activities of bacteria. In addition to playing a negative regulatory role in PG synthesis, Fnr regulators also regulate carbapenem biosynthesis and flagellar genes. For the former, Fnr negatively regulates carbapenem biosynthesis through CarR, a gene that can encode a pathway specific activator of bacteriostatic carbapenems; For the latter, Fnr can enhance the expression of flagellar genes, which is conducive to bacterial motility. Therefore, it can be seen that knocking out or modifying a gene has both advantages and disadvantages for the bacteria, and the appropriate method should be selected according to the actual situation (Sun et al., 2021). In one study, researchers introduced clusters of biosynthetic genes encoding PGs into *Pseudomonas putida* and used genome editing tools to make auxiliary proteins required for polyketide synthases (PKSs) and NRPSs more available, leading to increased PG production (Cook et al., 2021).

Secondly, we can introduce genes related to PG production into suitable host cells for mass production. The ligase encoded by *pigC* is the key enzyme that links MAP and MBC, and the synthesis rate and amount of this enzyme are important for PG production. Researchers introduced the *pigC* gene into *E. coli*, which greatly increased the expression of *E. coli pigC* through self-induction and a good induction strategy, as well as increased PG production due to *pigC*’s function of linking MAP and MBC (You et al., 2018).

In terms of optimizing cultivation conditions, a large number of researchers have conducted research from aspects such as medium composition, production conditions, and microbial species. Culture conditions that result in a relatively high increase in PG yield are summarized in Table 2. Before large-scale synthesis, optimal production conditions are typically determined experimentally in the laboratory, encompassing the identification of the optimal temperature, pH, carbon and nitrogen sources, and their suitable ratio (Kurbanoglu et al., 2015). Researchers have conducted extensive studies on the media for PG production, starting with commercial media such as nutrient broth, Luria-Bertani medium, and peptone-glycerol broth. Adding raw materials for the synthesis of MBC and MAP to these media can enhance PG synthesis, such as proline and fatty acids. Later on, researchers found that there is a great potential for the production of medium composed of inexpensive agricultural and fishery by-products, such as peanut seed, peanut oil (Giri et al., 2004), potato starch, cassava (de Araujo et al., 2010), brown sugar (Aruldass et al., 2014), crab shells, squid pens (Nguyen et al., 2019), and shrimp shells (Nguyen et al., 2021). These high-yield, low-cost, and environmentally significant substrates are considered the focus of large-scale PG synthesis (Bhagwat and Padalia, 2020; Wang et al., 2020). For example, in the production of PG using Tannery Fleshing, the yield was around 47 mg/mL under optimum production conditions (Sumathi et al., 2014); the highest yield of 27.65 mg/mL was obtained when using soybean oil as a carbon source and peptone as a nitrogen source with a C/N of 100/10 (Liu et al., 2021).

**Table 2:** Summary of the optimization of production conditions.

Strains	Medium composition	Temperature (°C)	PH	Yield (mg/L/h)	References
*S. marcescens* UCP 1549	Solid medium; 6% cassava wastewater supplemented with 2% mannitol	28	7	1031.25 PG	de Araujo et al. (2010)
*S. marcescens* UCP 1549	SSF; WB impregnating salts solution containing 5% WSO	28	ND	499.17 PG	Dos Santos et al. (2021)
*S. marcescens* NPLR1	Fermentor; Salt solution containing 3% Tannery Fleshing	30	8	1175.00 PG	Sumathi et al. (2014)
*S. marcescens* WT	Erlenmeyer flask; 2% powdered peanut seed in distilled water	28	7	1076.38 PG	Giri et al. (2004)
*S. marcescens* WT	Erlenmeyer flask; 1 g/L potato starch powder, 0.6 g/L casein and 1 g/L yeast extract	30	7	100.00 PG	Suryawanshi et al. (2014)
*H. chejuensis* (KCTC 2396)	Bioreactor; marine broth	25	6.89	452.38 PG	Jeong et al. (2021)
*S. marcescens* TKU011	Erlenmeyer flask; 1% α-chitin, 0.6% casein, 0.05% K_2_HPO_4_, and 0.1% CaSO_4_	25	5.65–6.15	96.25 PG	Nguyen et al. (2019)
*S. marcescens* FZSF02	Erlenmeyer flask; glucose, peanut powder, beef extract, and olive oil	26	7	214.20 PG	Lin et al. (2019)
*S. marcescens* C3	Erlenmeyer flask; starch/peptone = 16 g/L/10.67 g/L, trace elements and alginate beads.	30	7	216.67 PG	Chen et al. (2013)
*S. marcescens* TNU01	Bioreactor; 1.12% de-SSP/0.48% casein and 0.02% K_2_SO_4_ and 0.05% K_2_HPO_4_	27	7	775.00 PG	Nguyen et al. (2021)
*S. marcescens* TNU02	Bioreactor; 1.12% de-CSP/0.48% casein and 0.02% (NH4)_2_SO_4_, 0.1% K_2_HPO_4_	27	6.15	637.50 PG	Nguyen et al. (2020)
*S. marcescens* SS-1	Erlenmeyer flask; YE medium and 10 g/L Proline	30	8	104.17 UP	Wei et al. (2005)
*S. marcescens* WT	Erlenmeyer flask; powdered peanut seed broth	28	7	783.33 PG	Shahitha (2012)
*S. marcescens* UTM1	Bioreactor; 10% brown sugar	25	7	337.88 PG	Aruldass et al. (2014)
*S. marcescens* BWL1001	Erlenmeyer flask; Soybean oil 100 g/L, peptone 10 g/L	28	5	768.06 PG	Liu et al. (2021)

SSF, solid-state fermentation; WB, wheat bran; WSO, waste soybean oil; ND, data not available; WT, wild type; de-SSP, demineralized shrimp shell powders; YE, yeast extract; UP, undecylprodigiosin.

## 4 Discussion

The discovery of prodigiosin (PG) has sparked interdisciplinary research interest in its bioactivities, such as its chemical makeup, bioactivity mechanisms, synthetic pathways, regulation, and production optimization within the prodiginine family. The identification of new PG members has highlighted their widespread presence in nature, inspiring further investigation into their bioactivity and potential for large-scale production. The mechanism of PG bioactivity is a focus of research. The mechanisms responsible for the anti-cancer effects of PG are multifaceted and complex, involving various aspects such as cell proliferation, apoptosis, angiogenesis, signaling pathways, and antioxidation. The advantage of PG as a natural product lies in its relatively low-toxicity, making it a highly anticipated potential candidate for pharmaceutical development. Despite the potential therapeutic effects observed in cellular and animal models, further in-depth research and experiments are still required before clinical application. PG demonstrates broad-spectrum antimicrobial activity, effectively targeting various microorganisms including bacteria and fungi. This characteristic makes it a valuable tool in combating complex infectious pathogens. While some antibacterial mechanisms have been identified, further research is necessary to gain a detailed molecular-level understanding of PG’s mechanisms.

It is important to note that nanocomposite technology utilizing PG for disease treatment has become a popular area of research due to PG’s strong biological activity. Nanomaterials can address PG’s hydrophobicity and low bioavailability, overcome its limitations in disease treatment, and improve its ability to transport and release in the body, resulting in better therapeutic outcomes (Araujo et al., 2022; Majumdar et al., 2022). This area of nanocarrier delivery is relatively less explored compared to other research directions, and some novel nanoparticles such as dendrimer and PG can be investigated next to explore better therapeutic effects.

Since researchers have studied the gene clusters involved in direct PG synthesis and obtained different gene clusters for different strains, there has also been a great deal of research on transcriptional regulatory systems or factors that play important roles in PG synthesis, such as QS, TCSs, and other regulatory factors. However, we have a poor understanding of some of the regulatory mechanisms behind the effects of synthesis, for example, the exact mechanism of the effect of LuxS/AI-2 on PG synthesis is still unknown.

Regarding large-scale production, the expanding application range and increasing market demand for PG have led to research on optimized cultivation techniques, high-yielding microbial strains, and extraction and purification techniques. This research lays the foundation for the industrial production of PG. Regarding culture medium, the focus of research is on using inexpensive and high-yield agricultural by-products to produce PG in large quantities, which also has environmental significance. As for microbial strains, *S. marcescens* remains the most commonly used. It is worth mentioning that *S. marcescens* is an opportunistic pathogen and exposure to this bacterium in immunocompromised individuals can cause various diseases such as wound infections, eye infections, meningitis, septicemia, and others. In case of infection, antibiotics like ciprofloxacin and gentamicin can be taken (Zivkovic Zaric et al., 2023). There have been experiments to transform wild-type strains into stable and excellent strains, which can then be combined with high-quality culture medium for mass production.

## 5 Conclusion

In our comprehensive review, we delve into recent advancements in understanding the chemical structure, physicochemical properties, gene regulation, and optimized production techniques of PG, shedding light on the underlying mechanisms at play. We underscore the extraordinary biological activity of PG and the factors that impact its production efficiency. Despite significant progress, there are still numerous areas within the mechanistic studies that warrant further investigation. To deepen our comprehension of PG’s interactions with diverse pathogens, it is imperative to conduct thorough examinations into its toxicity profile, pharmacokinetics, drug compatibility, and other pertinent characteristics. Future research endeavors should prioritize exploring the synergistic potential of PG in combination with other therapeutic modalities and delineating their tailored applications across various cancer types and infections. With regards to large-scale production, optimizing culture conditions in alignment with elucidated regulatory mechanisms, selecting and enhancing high-yielding strains through exploring natural microorganisms or implementing mutagenesis and genetic engineering techniques, and ultimately integrating superior culture conditions with high-performing strains are pivotal steps toward achieving elevated production yields. These strategies are pivotal in laying a robust foundation for advancing PGs as potent anticancer and antimicrobial agents.

## Author contributions

YL: Visualization, Writing – original draft. DL: Visualization, Writing – original draft. RJ: Visualization, Writing – original draft. ZL: Writing – review & editing. XG: Conceptualization, Writing – review & editing, Supervision.
